# Polydopamine and peptide decorated doxorubicin-loaded mesoporous silica nanoparticles as a targeted drug delivery system for bladder cancer therapy

**DOI:** 10.1080/10717544.2017.1309475

**Published:** 2017-04-17

**Authors:** Yi Wei, Li Gao, Lu Wang, Lin Shi, Erdong Wei, Baotong Zhou, Li Zhou, Bo Ge

**Affiliations:** 1 Department of Urology, Affiliated Hospital of Guilin Medical University, Guilin, P.R. China,; 2 College of Biotechnology, and; 3 Pharmaceutical College, Guilin Medical University, Guilin, P.R. China

**Keywords:** Drug delivery, mesoporous silica, polydopamine, targeted therapy, bladder cancer

## Abstract

We reported a simple polydopamine (PDA)-based surface modification method to prepare novel targeted doxorubicin-loaded mesoporous silica nanoparticles and peptide CSNRDARRC conjugation (DOX-loaded MSNs@PDA-PEP) for enhancing the therapeutic effects on bladder cancer. Drug-loaded NPs were characterized in terms of size, size distribution, zeta potential, transmission electron microscopy (TEM), Brunauer–Emmett–Teller (BET) surface area and drug loading content. *In vitro* drug release indicated that DOX-loaded MSNs@PDA and MSNs@PDA-PEP had similar release kinetic profiles of DOX. The PDA coating well controlled DOX release and was highly sensitive to pH value. Confocal laser scanning microscopy (CLSM) showed that drug-loaded MSNs could be internalized by human bladder cancer cell line HT-1376, and DOX-loaded MSNs@PDA-PEP had the highest cellular uptake efficiency due to ligand–receptor recognition. The antitumor effects of DOX-loaded nanoparticles were evaluated by the MTT assay *in vitro* and by a xenograft tumor model *in vivo*, demonstrating that targeted nanocarriers DOX-loaded MSNs@PDA-PEP were significantly superior to free DOX and DOX-loaded MSNs@PDA. The novel DOX-loaded MSNs@PDA-PEP, which specifically recognized HT-1376 cells, can be used as a potential targeted drug delivery system for bladder cancer therapy.

## Introduction

Bladder cancer is one of the deadliest forms of cancer in modern medicine. Most cases of bladder cancer are superficial tumors that are confined to the epithelial cell layer of the bladder and are treated by transurethral resection (Hu et al., 2014; Jung et al., 2016). Nevertheless, the tumors that invade muscle layer of the bladder may spread to metastasis. Metastatic bladder cancer has been treated with chemotherapeutic drugs, but no higher than 15% of the patients show long-term disease-free survival (Dinney et al., 2004; Zhou et al., 2013). The most popular anticancer drugs, such as doxorubicin (DOX) and paclitaxel, have high toxicity and low specificity, leading to serious toxic side effects (Jaiswal et al., 2015; Zhang et al., 2015b; Zhu et al., 2016).

Nanotechnology is recognized as a revolutionary manufacturing technology involving multidisciplinary research issues that rely on the understanding and control of substances at the nanoscale length (Zhu et al., 2014; Beik et al., 2016; Mariam et al., 2016). Cancer nanotechnology, as an integrated platform, provides great opportunities for improving drug efficacy and pharmacokinetics to reduce side effects. Nanoparticles (NPs) with variable size, shape and surface modification have been exploited to achieve effective tumor vascular targeting (Zhang et al., 2015a; Hashemikia et al., 2016). Functional NPs suitable for nanomedicine are typified by encapsulation of drugs, prevention of recognition by macrophages, long circulation time in the bloodstream, and site-specific delivery with targeted moiety (Zeng et al., 2013; Tummala et al., 2016). The sizes of NPs, which ranged from 50 to 300 nm, allow extravasation and accumulation in tumor sites, also known as the enhanced permeability and retention effect (Attia et al., 2014; Lian et al., 2016). Passive targeting is based on pathophysiological characteristics specific to solid tumors, such as hypervascularity, potential secretion of vascular permeability factors, irregular vascular architecture, and absence of effective lymphatic drainage that prevents efficient clearance of large molecules (Acharya & Sahoo, 2011). Active targeting is primarily based on specific binding of receptors to ligands (e.g. aptamer and small-molecule folic acid) (Tao et al., 2015; Wu et al., 2017).

As novel nanotherapeutic tools, biocompatible, biodegradable, renewable and nontoxic mesoporous silica NPs (MSNs) have been used as drug vehicles for cancer treatment in past decades (Xiao et al., 2013; Ahmadi et al., 2014). MSNs have high surface areas and large pore volumes. Furthermore, they can readily be surface-functionalized. MSNs are applicable drug delivery systems after functionalization of the interior and exterior surfaces of mesoporous materials (Niedermayer et al., 2015; Niemela et al., 2015; Gu et al., 2016). Therefore, they have been employed for delivery of chemotherapeutic drugs, proteins and genes or co-delivery (Luo et al., 2014; Jiao et al., 2015; Maggini et al., 2016).

Drugs enter MSNs mainly through adsorption, so the latter must be surface-modified to prevent drug leakage and to control drug release. Currently, many materials, such as ZnO or Fe_3_O_4_ NPs, copolymers and cyclodextrin-based assemblies, have been used as gatekeepers for drug release from MSNs (Zhu et al., 2016). Despite these general prosperities, most of the MSNs gatekeepers still have critical problems such as the scarcity of biocompatibility and unpredictable toxicity. Therefore, a novel capping agent as gatekeeper with low or no potential risks and could also respond to internal biological microenvironment is highly anticipated. Polydopamine (PDA) is a biomimetic polymer which can form on a wide range of materials including ceramics, copolymers and semiconductors through oxidative polymerization in a weak alkaline condition (pH 8.0–8.5) (Park et al., 2014; Das & Jana, 2015; Chang et al., 2016). PDA coating, as an excellent gatekeeper on the surface of MSNs, is extremely sensitive to pH value. With PDA coating, drug molecules are easily blocked in MSNs under neutral condition and released at lower pH values (Zheng et al., 2014; Cho & Kim, 2015). Functional ligands contain nucleophilic functional groups such as amine and thiol which can be bioconjugated onto surface PDA coating via Michael addition or Schiff base reactions (Lee et al., 2007a, 2009; Meng et al., 2015). Being both simple and versatile, this method has been widely used to functionalize various types of substrates since its discovery in 2007 (Lee et al., 2007a; Park et al., 2014).

Targeted drug delivery system, which can carry drugs to specific tissues or organs, has been applied in cancer nanotechnology (de Oliveira et al., 2016; Ulbrich et al., 2016). Screening of phage displayed peptide libraries has been used to confirm peptides that selectively target to cancer cells or target proteins which are on the surface of cell (Wang et al., 2010; Jia et al., 2012; Key et al., 2016). Lee’s group reported that Bld-1 peptide CSNRDARRC could target to bladder cancer cells (such as HT-1376 and T-24 cells) through the way of phage display (Lee et al., 2007b; Jung et al., 2016). Thus, drug-loaded NPs which conjugate peptide CSNRDARRC can specifically recognize bladder cancer cells for targeted cancer therapy.

In this study, DOX-loaded PDA-modified MSNs (DOX-loaded MSNs@PDA) were prepared for bladder cancer treatment. Then CSNRDARRC, as a targeting peptide moiety, was bioconjugated on the PDA coating of DOX-loaded MSNs@PDA. Their particle size, size distribution, zeta potential, surface morphology, Brunauer–Emmett–Teller (BET) surface area, drug loading content (LC) and *in vitro* drug release profiles were analyzed. MTT assay using bladder cancer cells HT-1376 and confocal laser scanning microscopy (CLSM) were employed to study the *in vitro* cytotoxicity and cellular uptake of DOX-loaded NPs. Furthermore, the *in vivo* antitumor efficacy of the prepared NPs on HT-1376 cell-bearing nude mice was also evaluated.

## Materials and methods

### Materials

Tetraethylorthosilicate (TEOS), cetyl trimethyl ammonium bromide (CTAB), ammonium fluoride (NH_4_F), dopamine hydrochloride and DOX hydrochloride were purchased from Sigma-Aldrich (St. Louis, MO). Targeted ligand peptide CSNRDARRC sequence was synthesized and validated by ChinaPeptides Co., Ltd. (Shanghai, China). Besides HPLC-grade chromatographic solvents, the other chemicals all had the highest commercially available grade. Distilled deionized water was utilized throughout this study. Human bladder cancer cell line HT-1376 was purchased from American Type Culture Collection (ATCC, Rockville, MD).

### Synthesis of MSNs

MSNs were synthesized according to a previously reported method (Hu & Cui, 2012; Chang et al., 2016). In brief, CTAB (1.82 g, 5 mmol) and NH_4_F (3 g, 81 mmol) were dissolved in 500 mL of water and heated up to 80 °C. Under vigorous stirring, TEOS (9 mL, 8.41 g) was added dropwise to the mixture solution that was then kept at 80 °C for 6 h. The solid product was centrifuged (12 000 rpm, 10 min), washed with water and ethanol several times and dried at 40 °C for 24 h under vacuum. To remove the surfactant CTAB, the resulting MSNs were dispersed in 400 mL of ethanol containing 8 mL of hydrochloric acid (37%) and refluxed at 80 °C for 24 h (Gao et al., 2012). The technique was repeated three times to make sure the surfactant was fully removed. The obtained MSNs were centrifuged, washed with deionized water, and finally dried under vacuum for 24 h.

### Drug loading and PDA surface modification

For the loading of anticancer drug DOX–HCl, 100 mg MSNs were added to a water solution of DOX–HCl (5 mL, 10 mg/mL) and stirred for 24 h. Thereafter, the DOX-loaded MSNs were centrifuged and washed twice with neutral deionized water before being dried at 40 °C under vacuum. All the washings including those before capping were collected, and the drug LC was evaluated from the difference between the concentration of initial DOX solution and that of the reaction medium combined with subsequent washings. DOX was analyzed by UV/Vis spectroscopy at the wavelength of 480 nm. For the surface modification with PDA, 50 mg DOX-loaded MSNs were dispersed in 50 mL of Tris–HCl buffer (pH 8.5, 10 mM) and then 25 mg dopamine hydrochloride was added. The mixture was stirred in dark at room temperature for 5 h. Then, PDA-coated drug-loaded MSNs were centrifuged and washed several times with deionized water to remove the unpolymerized dopamine (Zheng et al., 2014). The surface-modified products were designated as DOX-loaded MSNs@PDA. Blank samples (drug-free MSNs) were also prepared by the same method.

### Conjugation of targeted peptide to PDA-coated MSNs

The functional targeted ligand peptide (PEP) was conjugated to the surface of DOX-loaded MSNs@PDA *via* the Michael addition reaction. In brief, MSNs@PDA were resuspended in Tris buffer (10 mM, pH 8.5) containing PEP. The concentrations of MSNs@PDA and ligand were 5 and 1 mg/mL, respectively. After 3 h of incubation at room temperature with stirring, the resultant NPs which were designated as MSNs@PDA-PEP were centrifuged, washed three times with deionized water and then dried under vacuum. Dopamine polymerization included brief incubation of the preformed MSNs@PDA in a weak alkaline solution of dopamine, followed by secondary incubation with amine-terminated functional ligands in aqueous solution via the Michael addition reaction. Using this method, we functionalized MSNs@PDA with PEP as the targeted ligand.

### Characterizations of NPs

The size and zeta potential of MSNs were measured by Malvern Mastersizer 2000 (Zetasizer Nano ZS90, Malvern Instruments Ltd., Malvern, UK). Freshly prepared MSNs were diluted appropriately before measurements that were performed at room temperature after 10 min of equilibration. The average of three independent measurements was recorded. As to transmission electron microscopy (TEM, Tecnai G2 20, FEI, Hillsboro, OR), MSNs were dropped onto a carbon membrane-coated copper grid. Before characterization, the grid was allowed to dryness. Nitrogen (N_2_) adsorption/desorption isotherms were measured at –196 °C by an ASAP 2020 accelerated surface area and porosimetry system (Micromeritics, Norcross, GA). The surface areas were calculated by the BET method, and the pore sizes and volumes were evaluated following the Barrett–Joyner–Halenda (BJH) method. Thermal gravity analysis (TGA) was conducted with Netzsch STA 449 (Selb, Germany) in an O_2_ atmosphere by heating the sample to 800 °C at the rate of 20 °C/min. The changes of the chemical composition in the surfaces of MSNs after modification with PDA and targeted ligand PEP were analyzed by Fourier transform infrared (FT-IR) spectroscopy (Nicolet, San Carlos, CA) with a pellet of powdered KBr. X-ray photoelectron spectroscopy (XPS) (Kratos Ltd., Manchester, UK) was carried out with an AXIS His spectrometer using a monochromatic Al Kα X-ray source (1486.6 eV photons, 150 W). Binding energies were referenced to the Fermi edge, and charge correction was performed by setting the C1s peak at 284.8 eV.

### *In vitro* drug release kinetic profiles

*In vitro* drug release from DOX-loaded NPs was tested as described before (Wang et al., 2015a). Briefly, 5 mg dried DOX-loaded MSNs, DOX-loaded MSNs@PDA and DOX-loaded MSNs@PDA-PEP were dispersed in 1 mL of PBS at different pH values (7.4, 6.0 and 5.0). Then the dispersion was transferred into a dialysis bag (MWCO = 3500, Spectra Por®, Spectrum, Houston, TX), and the bag was immersed into a centrifuge tube containing 10 mL of PBS and stirred at 37 °C. The tube was thereafter placed in an orbital shaker water-bath and shaken at 37 °C and 120 rpm. At designated time intervals, the full release buffer was collected and replaced with 10 mL of fresh PBS (kept at the same temperature). Released drug DOX was quantified by UV/Vis spectroscopy at the wavelength of 480 nm. The relationship between accumulative DOX release from drug-loaded MSNs and time was plotted.

### Cell culture

In a humidified 5% CO_2_ atmosphere, HT-1376 cells were cultured at 37 °C in Dulbecco's Modified Eagle's Medium (DMEM) supplemented with 1% penicillin/streptomycin and 10% fetal bovine serum.

### Cellular uptake of DOX-loaded NPs

HT-1376 cells were incubated with free DOX, DOX-loaded MSNs@PDA and DOX-loaded MSNs@PDA-PEP (equivalent DOX concentration of 5 μg/mL) for 2 h at 37 °C, washed three times with cold PBS, and fixed for 20 min in methanol. Afterwards, the cells were washed with PBS, stained with 4′,6-diamidino-2-phenylindole (DAPI) for 30 min, washed with PBS twice and finally observed using CLSM (LSM 410, Zeiss, Jena, Germany) equipped with an imaging software. The images of HT-1376 cells were captured with differential interference contrast channel, blue channel (DAPI) excited at 358 nm and red channel (DOX) excited at 543 nm (Zheng et al., 2011,2013). Furthermore, cellular uptake efficiency was quantified at the DOX concentrations of 1, 5 and 10 μg/mL.

### *In vitro* cytotoxicity using MTT assay

The viability of HT-1376 cells in the presence of free DOX and DOX-loaded NPs was evaluated by employing the MTT assay. HT-1376 cells were inoculated at the density of 5 × 10^3^ cells/well in 96-well plates and incubated in 100 μL of medium overnight. Then they were incubated by suspensions of free DOX, DOX-loaded MSNs, DOX-loaded MSNs@PDA and DOX-loaded MSNs@PDA-PEP at equivalent DOX concentrations of 0.2, 1, 5 and 10 μg/mL for 24 and 48 h, respectively. The formulations were replaced by 5 mg/mL MTT-containing DMEM at indicated time intervals. After incubation of the cells for 4 h, MTT was aspirated off and formazan crystals were dissolved by adding DMSO. The absorbance was detected by a microplate reader (Bio-Rad Model 680, Watford, UK) at 570 nm, and calibrated to zero by using untreated cells with 100% viability as control and the cells without MTT addition as blank. The IC_50_ value, the drug concentration at which there is 50% inhibition of cell growth compared to the control sample, was calculated by the *in vitro* cytotoxicity data. Similar experiments were performed on human embryonic kidney HEK 293 cells (ATCC, Rockville, MD) to further evaluate the safety of drug-free nanoparticles.

### *In vivo* antitumor efficacy of DOX-loaded NPs

Male severe combined immunodeficient mice (15–20 g, 5 weeks old) were bought from Institute of Laboratory Animal Sciences, Chinese Academy of Medical Science. All the protocols for the proposed *in vivo* experiments were approved by the Administrative Committee on Animal Research in the Guilin Medical University. Each mouse was subcutaneously injected with 100 μL of medium PBS containing about 2 × 10^6^ human bladder cancer cell HT-1376 into the back. The subcutaneous tumor growth after inoculation of HT-1376 cells was frequently observed. Tumor length and width were measured with a vernier caliper, and the three-dimensional volume of an ellipsoid tumor tissue (*V*) was calculated according to 4π/3 × (length/2) × (width/2)^2^ (Cao et al., 2015). Treatment was initiated when the volume reached approximately 80 mm^3^ (designated as the 0th day). The tumor-bearing mice were divided into four groups randomly (*n* = 5) and subjected to tail intravenous injection with saline (control), DOX, DOX-loaded MSNs@PDA and DOX-loaded MSNs@PDA-PEP in PBS at the dose of 10 mg DOX/kg on the 0th, 4th, 8th and 12th days, respectively. The tumor size and body weight of each mouse were measured every two days. After 16 days of treatment, the antitumor activity was evaluated by measuring the terminal tumor volume. In order to investigate the safety profile of DOX-loaded nanoparticles, the histological analysis was performed. Tumors and major organs (heart, liver, spleen, lung and kidney) were excised and fixed in 10% neutral buffered paraformaldehyde overnight. Thereafter, the tissues were embedded in paraffin and stained with hematoxylin and eosin (H&E) and observed by optical microscope.

### Statistical methodology

Unless stated otherwise, all experiments were carried out at least three times. All the experimental data are expressed as mean ± SD. Statistical analysis was performed by one-way ANOVA followed by the Bonferroni test with SPSS 16.0 software. Probability (*p*) less than 0.05 was considered statistically significant.

## Results and discussion

### Synthesis and characterizations of NPs

#### Particle size, zeta potential, drug LC, TEM morphology and BET surface area

**Scheme 1.**
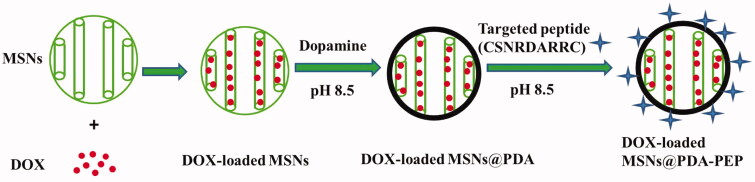
Schematic representation of the preparation techniques of DOX-loaded MSNs@PDA-PEP.

The MCM-41 type MSNs were first synthesized according to a previously reported method (Chang et al., 2016). Then, MSNs were surface modified by PDA. In a weak alkaline condition (pH 8.0–8.5), dopamine undergoes oxidative polymerization in the presence of oxygen as the oxidant. During dopamine polymerization, the dopamine catechol is oxidized to quinone, which reacts with other catechols and/or quinones to form PDA. Then, a coating layer PDA tightly adheres on the surface of MSNs which are immersed in the dopamine solution (Lee et al., 2007a; Jiang et al., 2011). The MSN suspensions turned dark after 5 h of reaction with dopamine at room temperature, indicating that dopamine was subjected to oxidative self-polymerization. Then, the targeted ligand PEP was successfully conjugated to the surface of DOX-loaded MSNs@PDA via the Michael addition reaction (Key et al., 2016; Tao et al., 2016). The surface modification of DOX-loaded MSNs by using oxidative self-polymerization of dopamine and ligand PEP is shown in Scheme 1. Moreover, the mechanism of polymerization of dopamine and the Michael addition reaction of PDA with the ligand PEP is presented in Figure S1.

The particle size and size distribution of NPs were detected by dynamic light scattering (DLS) (Table 1). The surface properties and particle sizes of NPs crucially participate in drug release, cellular uptake together with biodistribution and pharmacokinetics *in vivo* (Wang et al., 2013; Ru et al., 2016). The particle sizes of DOX-loaded MSNs@PDA and DOX-loaded MSNs@PDA-PEP were 168.3 ± 8.1 and 170.2 ± 7.5, respectively, which were larger than that of MSNs because of PDA coating. The zeta potentials of all NPs were negative, implying they were stable *in vivo* through electrostatic repulsion, as a prerequisite for drug delivery. The drug LC of DOX-loaded MSNs@PDA-PEP (16.25%) was a little lower than those of DOX-loaded MSNs and DOX-loaded MSNs@PDA, demonstrating drug molecules were barely lost during the preparation of targeted drug vehicles.

**Table 1.** Characterization parameters of MSNs, DOX-loaded MSNs, DOX-loaded MSNs@PDA and DOX-loaded MSNs@PDA-PEP.

Sample	Particle size^a^ (nm)	Zeta potential (mV)	Drug loading content (%)	BET surface area (m^2^/g)	Pore volume^b^ (cm^3^/g)	Pore size^c^ (nm)
1	124.6 ± 7.3	–22.8 ± 3.7	N/A	274.82	0.56	2.58
2	125.1 ± 6.4	–14.7 ± 2.4	16.61	97.51	0.29	1.46
3	168.3 ± 8.1	–17.3 ± 3.1	16.48	42.96	0.14	N/A
4	170.2 ± 7.5	–15.9 ± 1.6	16.25	36.55	0.12	N/A

1: MSNs; 2: DOX-loaded MSNs; 3: DOX-loaded MSNs@PDA; 4: DOX-loaded MSNs@PDA-PEP.

N/A: not applicable.

^a^ NPs size was measured by dynamic light scattering.

^b^ BJH cumulative pore volume for pores between 1.7 and 300 nm in width.

^c^ Most probable pore size.

**Figure 1.**
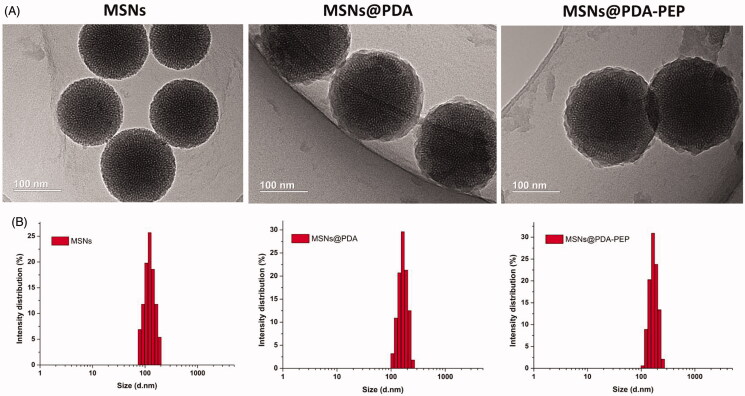
(A) TEM images and (B) DLS size distribution of MSNs, MSNs@PDA and MSNs@PDA-PEP.

TEM images (Figure 1A) show that MSNs are sphere-shaped and coated by a layer of polymer after surface modification with PDA. The image of PEP-modified MSNs is basically the same as that of MSNs@PDA. The size distributions of NPs detected by DLS are presented in Figure 1(B). Clearly, the particle sizes detected by TEM and DLS were consistent.

The BJH pore size distribution of MSNs is shown in Figure S2. The distribution of MSNs was rather narrow and the most probable pore size was 2.58 nm. The BET specific surface area, pore volume and most probable pore size of MSNs, DOX-loaded MSNs, DOX-loaded MSNs@PDA and DOX-loaded MSNs@PDA-PEP are displayed in Table 1. Compared with MSNs, all the pore parameters of drug-loaded MSNs and surface-modified NPs with PDA and PEP were significantly lower. Thus, anticancer drug DOX occupied the pore space of MSNs and DOX-loaded MSNs was coated with PDA.

#### Stability of DOX-loaded MSNs@PDA and DOX-loaded MSNs@PDA-PEP

During nanoformulation storage, drug-loaded NPs are prone to aggregation because the absolute value of zeta potential decreases. Therefore, their size distribution becomes non-uniform and only the fresh NPs are suitable for therapy. In this study, to investigate the stability of DOX-loaded MSNs@PDA and DOX-loaded MSNs@PDA-PEP (stored at 4 °C), their average size, size distribution and zeta potential were measured every 10 days until the 90th day after preparation. During three months of storage, the size or zeta potential of DOX-loaded MSNs@PDA and DOX-loaded MSNs@PDA-PEP hardly changed at 4 °C (Figure S3), demonstrating that all these NPs were fairly stable.

### TGA, FT-IR and XPS results

**Figure 2.**
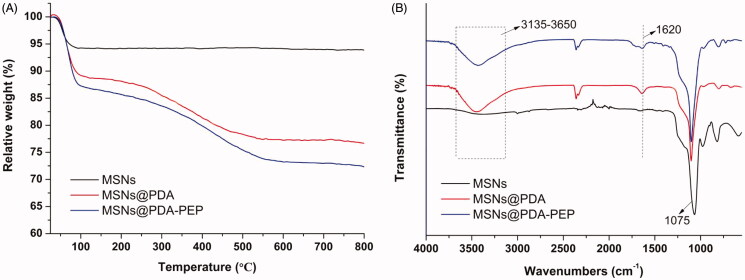
(A) TGA curves and (B) FT-IR spectra of MSNs, MSNs@PDA and MSNs@PDA-PEP.

The TGA curves are shown in Figure 2(A). The weight losses of MSNs@PDA and MSNs@PDA-PEP were 23.35% and 27.68% respectively when heated in an O_2_ atmosphere to 800 °C, while that of MSNs was only 6.13% in the same temperature range. The TGA results confirmed MSNs were successfully surface-modified with PDA and targeted ligand PEP. Moreover, the contents of PDA coating and ligand PEP were 17.22% and 4.33%, respectively (Nematollahzadeh et al., 2013). FT-IR spectra were used to characterize the surface chemical group composition of MSNs. As shown in Figure 2(B), several new absorption signals appeared after surface modification with PDA coating and targeted ligand PEP. The broad absorbance between 3135 and 3650 cm^−1^ were assigned to the stretching vibrations of N–H/O–H of PDA. The peak at 1620 cm^−1^ was corresponded to the C–C resonance vibrations in the aromatic ring. The peaks at 1515 and 1409 cm^−1^ might result from the targeted ligand PEP. All the nanoparticles presented absorption peaks at 1075 cm^−1^, which were caused by Si–O–Si stretching vibrations on MSNs network (Abd-Elrahman et al., 2016; Zhu et al., 2016).

**Figure 3.**
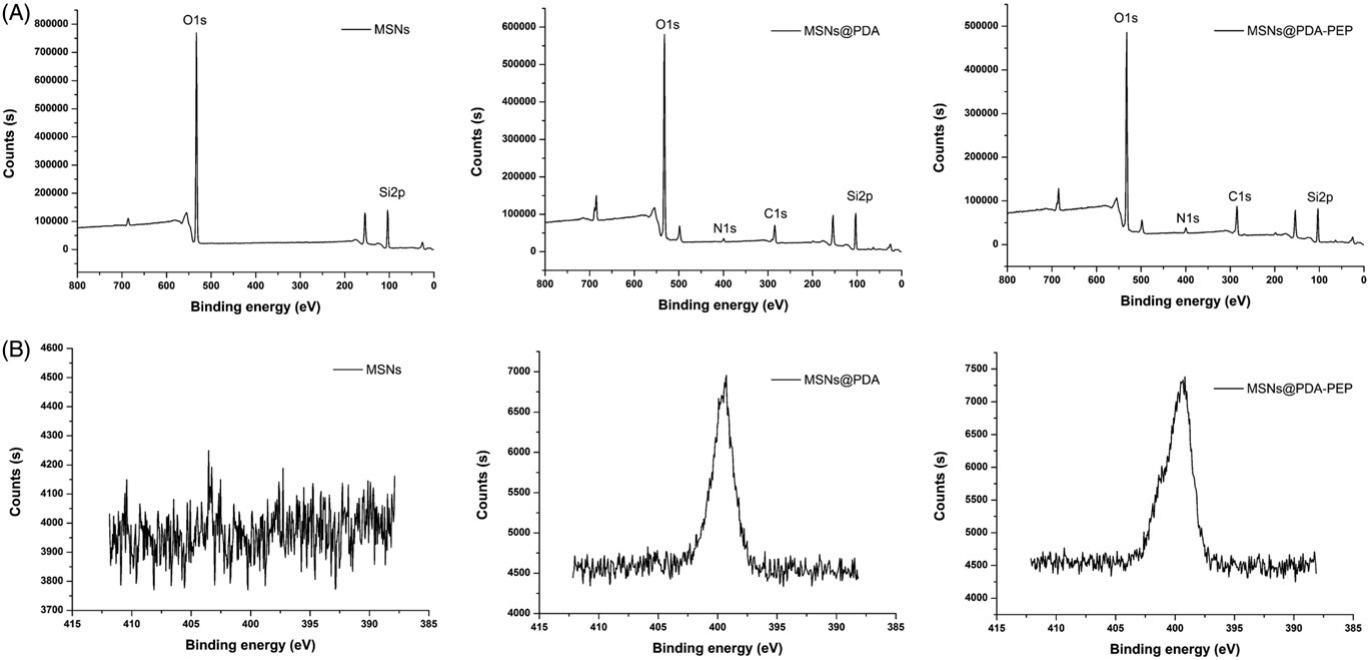
XPS spectra of MSNs, MSNs@PDA and MSNs@PDA-PEP. (A) Wide scan; (B) narrow scan for N1s peaks.

XPS further provided information with higher sensitivity for determining the surface chemical composition. The binding energies of 397–403 eV in the N1s spectra of MSNs@PDA and MSNs@PDA-PEP verified the presence of a PDA layer onto the precursor MSNs after modification, but the unmodified MSNs did not exhibit such peaks (Figure 3) (Zheng et al., 2014). The PDA coating and functionalization with PEP were also proven by narrow XPS scan for N1s peaks. As shown in Figure 3(B), N1s peaks appear after PDA modification. Furthermore, the N1s peaks of MSNs@PDA-PEP were more intense than those of MSNs@PDA, demonstrating the conjugation of targeted ligand PEP onto the surface of MSNs@PDA through the Michael addition reaction (Bernsmann et al., 2009; Liu et al., 2013). Taken together, TGA, FT-IR and XPS indicated that bare MSNs had been coated with PDA and PEP had been conjugated onto the PDA-modified MSNs surface successfully.

### Drug release kinetic profiles *in vitro*

**Figure 4.**
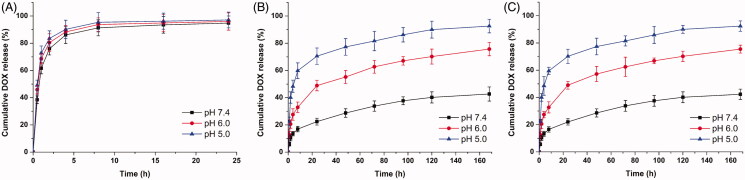
(A) *In vitro* drug release kinetic profiles of (A) DOX-loaded MSNs; (B) DOX-loaded MSNs@PDA; (C) DOX-loaded MSNs@PDA-PEP at different pH values.

*In vitro* release kinetics of drug-loaded NPs can be used to simulate the release process *in vivo* and to assess the effects of pH on drug release as well. The accumulative drug release kinetics curves of DOX-loaded MSNs, DOX-loaded MSNs@PDA and DOX-loaded MSNs@PDA-PEP in PBS at different pH values are shown in Figure 4, and those of the former two differ distinctly. At various pH values, the drug release from DOX-loaded MSNs was basically identical. Under neutral or acidic conditions, 60–70% of DOX was rapidly released within 1 h, and almost all of the drug was released within 8 h. In contrast, the drug release from DOX-loaded MSNs@PDA was obviously pH-dependent, which accelerated with decreasing pH. The release amount, which was as low as 22.4% at pH 7.4 within 24 h, was only 42.7% even in seven days. Under acidic conditions, however, the release amounts reached 48.9% and 70.5% within 24 h at pH 6.0 and 5.0, respectively. Accordingly, drug in the NPs without PDA modification freely diffused into solvent through pore channels, whereas PDA coating effectively prevented rapid drug release (Habibi et al., 2015). This could be attributed to the PDA coating was partially hydrolyzed and detached from the surface of MSNs in the acidic condition. Furthermore, the solubility of DOX is dramatically enhanced at low pH, which may also contribute to rapid release in mildly acidic buffers (Pranatharthiharan et al., 2016). In a word, PDA, as an outstanding gatekeeper, effectively encapsulated drug inside MSNs and prevented burst release from the pore channels. Therefore, the PDA-modified MSNs can be utilized as a drug controlled-release system. Since DOX-loaded MSNs@PDA-PEP and DOX-loaded MSNs@PDA had similar drug release kinetics curves, DOX was rarely leaked after conjugation with targeted ligand PEP. With extremely high pH responsiveness, PDA-modified NPs allowed controlled drug release at low pH values. As a result, drug was released only after endocytosis by tumor cells into lysosomes, which effectively reduced drug waste as well as boosted the antitumor effects by quickly elevating drug concentration in lysosomes.

### Cellular uptake of NPs

**Figure 5.**
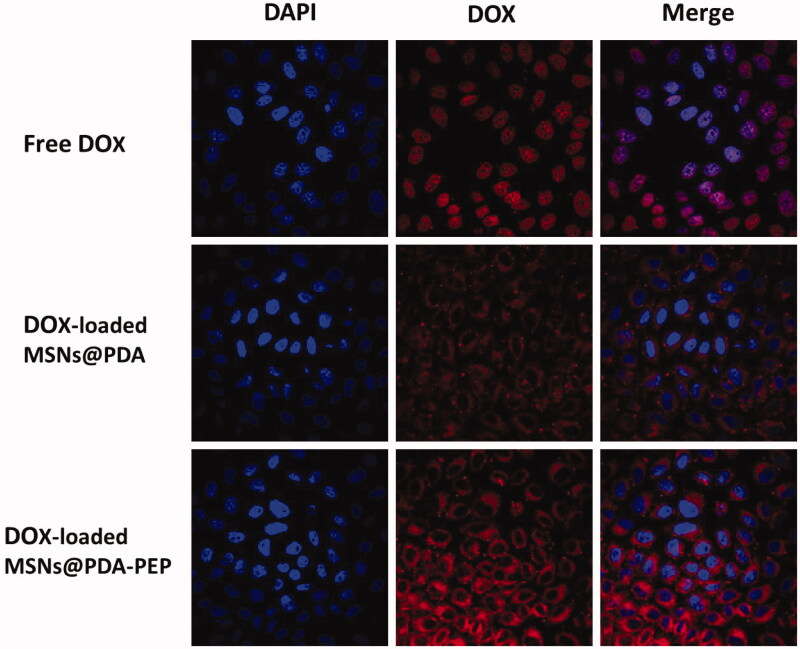
(A) CLSM images of HT-1376 cells incubated with free DOX, DOX-loaded MSNs@PDA and DOX-loaded MSNs@PDA-PEP for 2 h. The cells were stained by DAPI (blue) and drug DOX was red.

**Figure 6.**
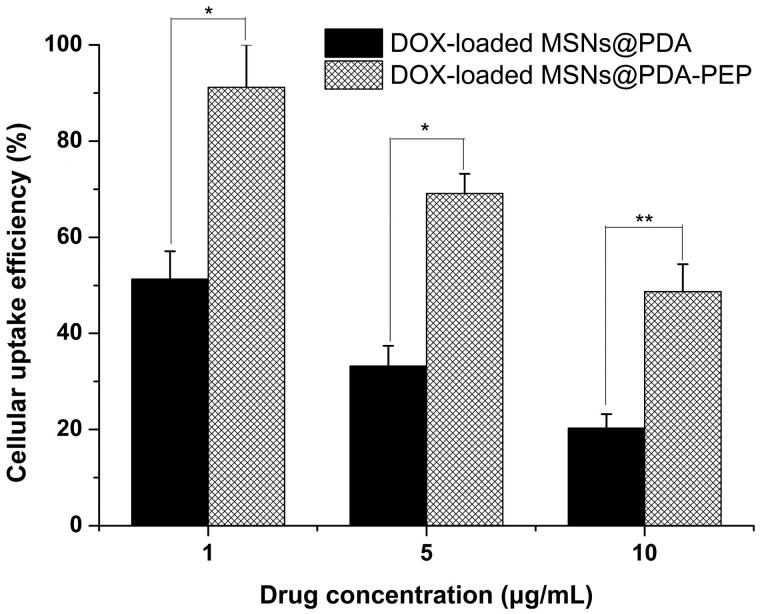
Cellular uptake efficiencies of DOX-loaded MSNs@PDA and DOX-loaded MSNs@PDA-PEP at different drug concentrations (*n *=3). *p < 0.05; **p < 0.01.

Usually, drug-loaded NPs exert therapeutic effects on cancer cells depending on internalization and sustained retention (Loureiro et al., 2016). *In vitro* investigation provides indirect evidence for verifying the advantages of nanoformulation over free anticancer drug, although it is different from the actual *in vivo* bioprocess. In this study, the cellular uptake of DOX, DOX-loaded MSNs@PDA and DOX-loaded MSNs@PDA-PEP was observed. Figure 5 presents the CLSM images of HT-1376 cells after they were incubated with the suspensions of DOX and DOX-loaded NPs in DMEM for 2 h (DOX concentration: 5 μg/mL). The images were captured from DAPI channel (blue) excited at 358 nm, DOX channel (red) excited at 543 nm and the merged one. Free DOX was mainly located in cell nuclei after uptake. However, DOX-loaded MSNs@PDA and DOX-loaded MSNs@PDA-PEP were located closely surrounding the blue cell nuclei by endocytosis, indicating these NPs could be effectively internalized into the cytoplasm of HT-1376 cells. Moreover, more drug-loaded NPs underwent cellular uptake after conjugation with PEP, suggesting that PEP specifically recognized and evidently targeted HT-1376 cells, which was of great significance to targeted bladder cancer therapy. Figure 6 shows the quantified uptake efficiencies of drug-loaded NPs. At DOX concentrations of 1, 5 and 10 μg/mL, the cellular uptake efficiencies of DOX-loaded MSNs@PDA-PEP were 1.78-fold, 2.08-fold and 2.40-fold those of DOX-loaded MSNs@PDA respectively, further revealing that peptide CSNRDARRC apparently targeted bladder cancer.

### *In vitro* cytotoxicity using MTT assay

**Figure 7.**
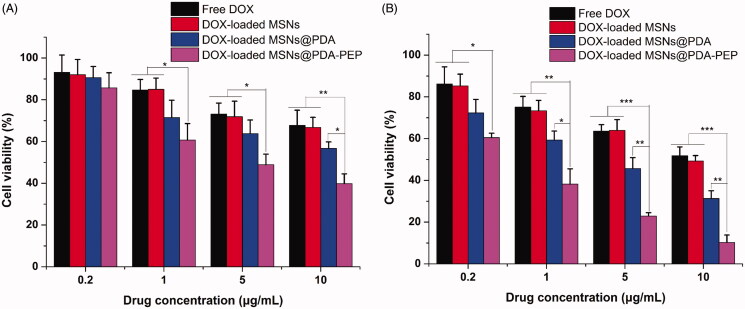
Viability of HT-1376 cells incubated with free DOX, DOX-loaded MSNs, DOX-loaded MSNs@PDA and DOX-loaded MSNs@PDA-PEP at the same drug concentrations: (A) 24 h; (B) 48 h (*n *=3). *p < 0.05; **p < 0.01; ***p < 0.001.

To evaluate the *in vitro* killing ability of drug-loaded NPs against bladder cancer, we calculated the cytotoxicity of free DOX, DOX-loaded MSNs, DOX-loaded MSNs@PDA and DOX-loaded MSNs@PDA-PEP against HT-1376 cells at drug equivalent concentrations of 0.2, 1, 5 and 10 μg/mL by the MTT assay. Figure 7 exhibits the relationships between cell survival rates at 24 h and 48 h and drug concentration, with untreated cells as the reference. The cytotoxicity of free DOX and drug-loaded NPs was enhanced with increasing DOX concentration and culture time. The cytotoxicity of free DOX almost resembled that of DOX-loaded MSNs unmodified with PDA. However, the cytotoxicity of DOX-loaded MSNs@PDA and DOX-loaded MSNs@PDA-PEP surface-modified with PDA surpassed that of free DOX, which can be attributed to the controlled release realized by PDA coating. Furthermore, DOX-loaded MSNs@PDA-PEP had the highest cytotoxicity, because targeted ligand PEP well specifically recognized and further killed HT-1376 cells potently. These results could be quantitatively demonstrated in terms of IC_50_ values. The IC_50_ values for HT-1376 cells after 24 and 48 h of incubation with free DOX, DOX-loaded MSNs, DOX-loaded MSNs@PDA and DOX-loaded MSNs@PDA-PEP are listed in Table 2. The IC_50_ values of DOX-loaded MSNs@PDA-PEP were 4.02 ± 0.58 and 0.46 ± 0.05 μg/mL after incubation 24 and 48 h, respectively, which were significantly lower than other DOX formulations. The IC_50_ values further indicated that the targeted DOX-loaded MSNs@PDA-PEP had better *in vitro* therapeutic effects on bladder cancer HT-1376 cell. Moreover, in order to evaluate the biocompatibility of DOX-free nanoparticles, the human embryonic kidney HEK 293 cells were employed for cytotoxicity research. Without loading DOX, all of the MSNs, MSNs@PDA and MSNs@PDA-PEP exhibited a negligible cytotoxicity against HEK 293 cells after 48 h at concentrations up to 500 μg/mL (Figure S4). These results suggested that the prepared materials and nanoparticles were basically nontoxic, highly biocompatible and safe (Park et al., 2014; Gao et al., 2015; Scott, 2015; Wang et al., 2015b).

**Table 2.** IC_50_ values of free DOX, DOX-loaded MSNs, DOX-loaded MSNs@PDA and DOX-loaded MSNs@PDA-PEP on HT-1376 cells following 24 and 48 h incubation, respectively.

	IC_50_ (μg/mL)			
Incubation time (h)	Free DOX	DOX-loaded MSNs	DOX-loaded MSNs@PDA	DOX-loaded MSNs@PDA-PEP
24	43.31 ± 2.65	43.75 ± 3.12	14.21 ± 1.16	4.02 ± 0.58
48	14.12 ± 1.33	12.67 ± 1.04	2.23 ± 0.32	0.46 ± 0.05

### *In vivo* antitumor efficacy

**Figure 8.**
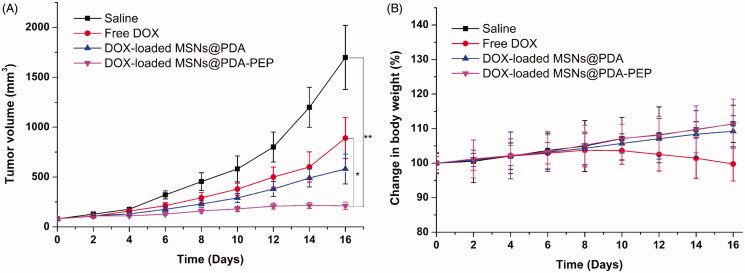
(A) Tumor growth and (B) percentage change of body weight curves of nude mice bearing HT-1376 cell xenograft after intravenous injection with saline, free DOX, DOX-loaded MSNs@PDA and DOX-loaded MSNs@PDA-PEP (*n *=5). *p < 0.05; **p < 0.01.

Due to the satisfactory cytotoxicity against HT-1376 cells *in vitro*, DOX-loaded MSNs@PDA-PEP may be an eligible vehicle for bladder cancer therapy. In the present study, the antitumor effects of DOX-loaded MSNs@PDA-PEP on HT-1376 cells-bearing nude mice were also assessed. Figure 8(A) presents the 16-day tumor growth of mice injected with control saline, free DOX, DOX-loaded MSNs@PDA and DOX-loaded MSNs@PDA-PEP. Every four days, the mice were subjected to tail intravenous injection with saline and drug for four consecutive cycles. Every two days, the tumor volume and body weight were recorded until the 16th day. Finally, the inhibitory effects followed a descending sequence of DOX-loaded MSNs@PDA-PEP > DOX-loaded MSNs@PDA > Free DOX > saline.

**Figure 9.**
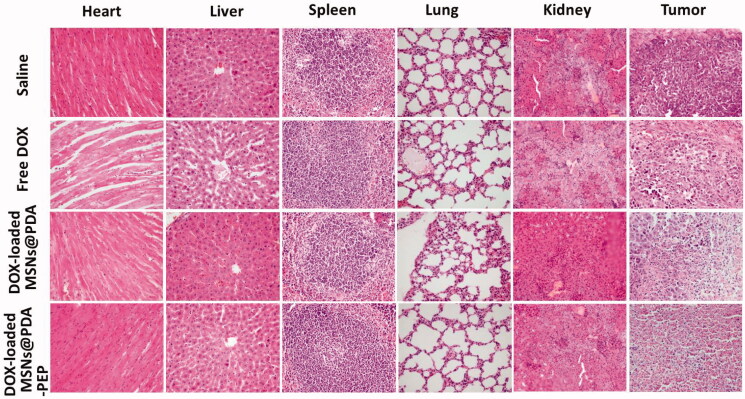
H&E analyses of tumors and major organs of nude mice bearing HT-1376 cell xenograft after treatments with saline, free DOX, DOX-loaded MSNs@PDA and DOX-loaded MSNs@PDA-PEP.

In the development of novel drug delivery systems, it is imperative to reduce the adverse events of drug-loaded NPs. The systemic adverse effects on animals can be evaluated by measuring body weight changes. Figure 8(B) presents changes in the body weights of all nude mice. The free DOX-treated group lost weight obviously, whereas the mice injected with DOX-loaded MSNs@PDA and DOX-loaded MSNs@PDA-PEP gained weight. Throughout the experiment, the mice treated by DOX-loaded MSNs@PDA-PEP remained vigorous and healthy, but those treated by free DOX exhibited weakened vitality. In order to further evaluate the safety and anticancer activity of DOX-loaded MSNs@PDA and DOX-loaded MSNs@PDA-PEP, tumors and organs (heart, liver, spleen, lung and kidney) were excised from mice on the 16th day and sectioned for H&E analyses and the results are presented in Figure 9. Treatments with DOX-loaded MSNs@PDA and DOX-loaded MSNs@PDA-PEP have not caused significant morphological changes in heart, liver, spleen, lung and kidney compared to the saline group. However, it is worth noting that the free DOX treated group had noticeable damage in heart and liver. The tumor tissues in DOX-loaded MSNs@PDA-PEP group showed significant tumor necrosis as compared to the saline, free DOX and DOX-loaded MSNs@PDA treated groups. Therefore, these results suggested that the targeted DOX-loaded MSNs@PDA-PEP were less susceptible to side effects and had the best antitumor effects on bladder cancer.

## Conclusions

A pH-sensitive and targeted drug delivery system of DOX-loaded MSNs@PDA-PEP was prepared successfully to enhance therapeutic effects on bladder cancer. The particle size of DOX-loaded MSNs@PDA increased after surface modification with PDA. The drug LC of DOX-loaded MSNs@PDA-PEP was a little lower than those of DOX-loaded MSNs and DOX-loaded MSNs@PDA, suggesting that DOX was hardly lost during the preparation of targeted drug vehicles. *In vitro* cellular uptake study indicated that DOX-loaded MSNs@PDA-PEP could be internalized by HT-1376 cells and had high targeting efficiency due to specifical recognition. Both *in vitro* cell experiments and *in vivo* animal studies demonstrated that the targeted NPs managed to enhance the therapeutic effects on bladder cancer compared with NPs without targeted functional modification and free DOX did. Histological analysis indicated that the targeted DOX-loaded MSNs@PDA-PEP had no obvious side effects on mice. Taken together, PDA-modified and peptide CSNRDARRC decorated NPs can be used as a potentially versatile drug delivery system for targeted bladder cancer therapy.

## Supplementary Material

Supplementary_Information.docx

## Notes

**Supplementary material available online**
