# Supplementary material for: Polydopamine and peptide decorated doxorubicin-loaded mesoporous silica nanoparticles as a targeted drug delivery system for bladder cancer therapy
Source: Drug Deliv. 2017 Apr 17;24(1):681–91. doi: 10.1080/10717544.2017.1309475 (PMC8241003; doi:10.1080/10717544.2017.1309475)
Supplement: Supplementary_Information.docx [file IDRD_A_1309475_SM2199.docx]

***Supplementary Information***

**Polydopamine and peptide decorated doxorubicin-loaded mesoporous silica nanoparticles as a targeted drug delivery system for bladder cancer therapy**

Yi Wei^a^, Li Gao^a^, Lu Wang^b^, Lin Shi^c^, Erdong Wei^a^, Baotong Zhou^a^, Li Zhou^a^, Bo Ge^a,*^

*^a^ Department of Urology, Affiliated Hospital of Guilin Medical University, Guilin, 541001, P.R. China*

*^b^ College of Biotechnology, Guilin Medical University, Guilin, 541004, P.R. China*

*^c^ Pharmaceutical College, Guilin Medical University, Guilin, 541004, P.R. China*

^*^Corresponding author

*E-mail address:* geboguilus@163.com (Bo Ge)

Tel./Fax: +86 773 2866955.





**Figure S1** Synthesis of peptide-conjugated PDA coating through oxidative polymerization and Michael addition reaction.

**
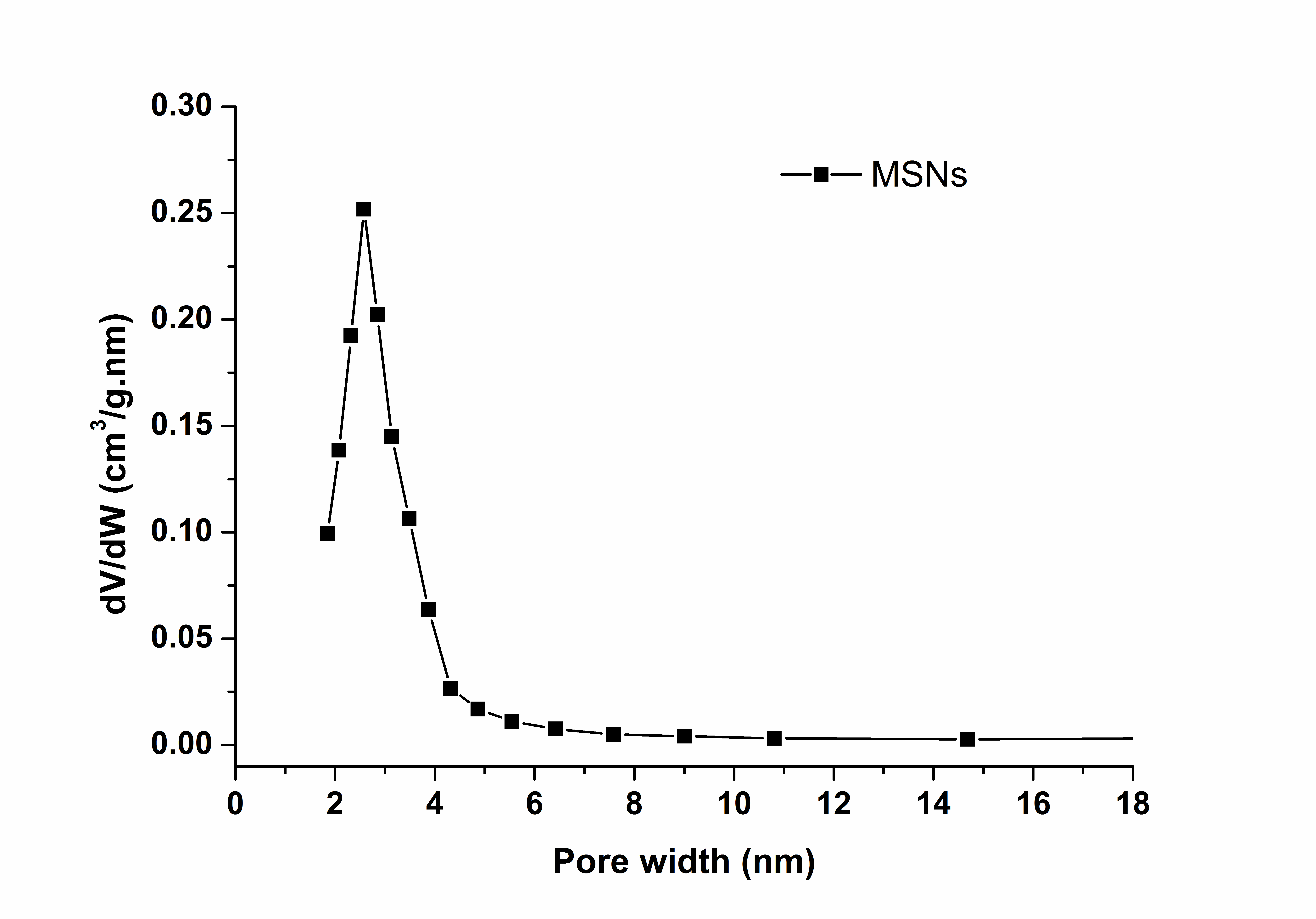
**

**Figure S2** Pore size distribution of MSNs from BJH adsorption.

**
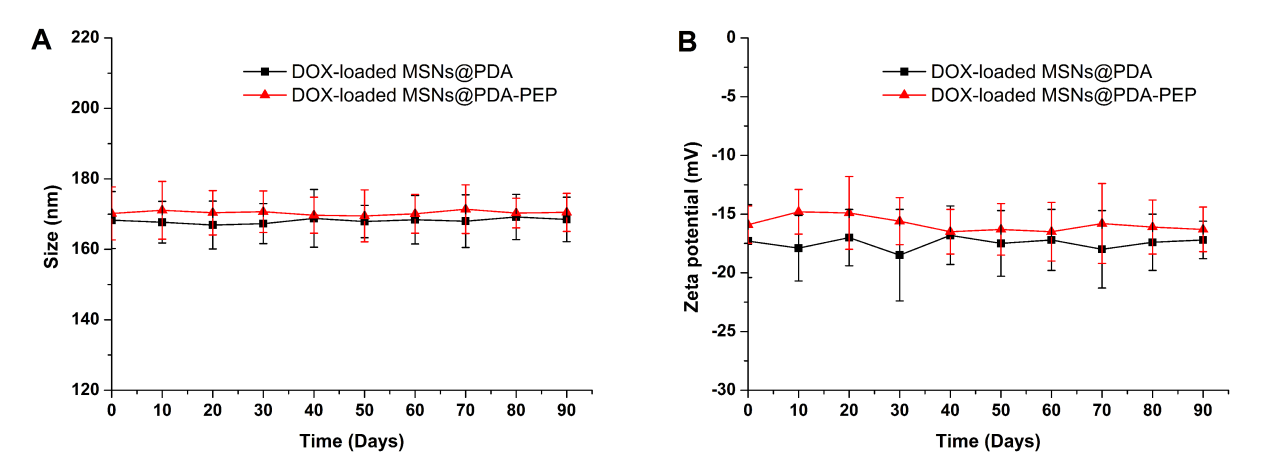
**

**Figure S3** *In vitro* stability of NPs: (A) Average sizes of DOX-loaded MSNs@PDA and DOX-loaded MSNs@PDA-PEP during 90 days of storage; (B) zeta potentials of DOX-loaded MSNs@PDA and DOX-loaded MSNs@PDA-PEP during 90 days of storage.





**Figure S4** *In vitro* cytotoxicity (HEK 293 cells) of drug-free MSNs, MSNs@PDA and MSNs@PDA-PEP test by MTT assay.
